# The Role of MicroRNAs in Repair Processes in Multiple Sclerosis

**DOI:** 10.3390/cells9071711

**Published:** 2020-07-16

**Authors:** Conor P. Duffy, Claire E. McCoy

**Affiliations:** School of Pharmacy and Biomolecular Sciences, Royal College of Surgeons in Ireland, 123 St Stephen’s Green, Dublin 2 D02 YN77, Ireland; conorduffymct@rcsi.ie

**Keywords:** autoimmune, CNS, experimental autoimmune encephalomyelitis, microRNA, multiple sclerosis, myelination, neuroinflammation, remyelination, therapies

## Abstract

Multiple sclerosis (MS) is an autoimmune disorder characterised by demyelination of central nervous system neurons with subsequent damage, cell death and disability. While mechanisms exist in the CNS to repair this damage, they are disrupted in MS and currently there are no treatments to address this deficit. In recent years, increasing attention has been paid to the influence of the small, non-coding RNA molecules, microRNAs (miRNAs), in autoimmune disorders, including MS. In this review, we examine the role of miRNAs in remyelination in the different cell types that contribute to MS. We focus on key miRNAs that have a central role in mediating the repair process, along with several more that play either secondary or inhibitory roles in one or more aspects. Finally, we consider the current state of miRNAs as therapeutic targets in MS, acknowledging current challenges and potential strategies to overcome them in developing effective novel therapeutics to enhance repair mechanisms in MS.

## 1. Introduction

### 1.1. Overview

Multiple sclerosis (MS) is an autoimmune disorder characterized by an attack on central nervous system (CNS) myelin sheath and subsequent demyelination, resulting in axonal damage, neuronal loss and the formation of numerous localized sclerotic lesions [1]. The condition manifests as disturbances in motor, sensory and cognitive function, and unlike many neurodegenerative disorders, the average age of onset is relatively young, at 30 years of age. The disease can be subtyped into three forms: relapse-remitting MS (RRMS), secondary progressive MS (SPMS) and primary progressive MS (PPMS). Approximately 85% of new diagnoses are RRMS, where abnormal peripheral immune invasion of the CNS leads to peripheral cell inflammatory attacks on the myelin sheath, most notably from activated T cells [2]. Following demyelination, bare axons are more vulnerable to further damage from reactive oxygen and nitrogen species, which infiltrating peripheral macrophages are thought to be the first mediators of [3]. RRMS is characterized by symptomatic ‘attacks’ (relapse) that later subside (remit) for periods of months or years. However, damage still tends to accumulate, and over time, RRMS will develop into SPMS in around 30% of cases at long-term follow up [4], where attacks are not followed by periods of remission and the condition becomes progressively worse. In PPMS, this phase of the disease appears from the onset, with no periods of remission and a steady accumulation of disability. This form of the disease affects approximately 15% of patients from the point of diagnosis [5]. In SPMS and PPMS, in contrast to RRMS, peripheral invasion of the CNS is far more limited, and most of the damage instead occurs ‘behind’ the blood–brain barrier (BBB). The mechanisms that mediate SPMS and PPMS damage are still not fully understood. However, it is likely to be some combination of accumulated inflammatory burden and neurodegeneration [6].

While treatments for MS are available, they tend to focus on limiting immune activity and infiltration, leading to less inflammatory damage to the CNS, and so primarily have efficacy in RRMS patients. There is a relative lack of treatments that target the progressive phases of the disorder and focus on repairing damaged lesions [7]. For this reason, understanding the biological processes that underlie myelin repair—remyelination—has become a research goal of considerable interest.

### 1.2. Remyelination

Following demyelination, the mammalian CNS naturally repairs and restores function via the process of remyelination. In remyelination, myelin debris is cleared by phagocytic cells like microglia and fresh myelin sheaths layered over bare neurons, formed by differentiation of recruited oligodendrocyte precursor cells (OPCs) into mature, myelinating oligodendrocytes [8,9]. Remyelination is a similar but physiologically distinct process to developmental myelination in several ways. Firstly, the surrounding microenvironment is importantly different. Developmental myelination occurring in tissues that are in the process of differentiating and establishing structure, while remyelination occurring in tissues that are developmentally mature but damaged and with other abnormal features such as peripheral immune infiltration. Secondly, the resultant myelin sheaths formed by remyelination are thinner and have a shorter internodal distance than those formed during developmental myelination [8,10].

The remyelination process via OPC differentiation is well characterized and dependent on several sequential steps, and has been comprehensively reviewed elsewhere [8]. Briefly, OPCs must be recruited and migrate to the point of demyelination and damage. This requires the OPCs to be ‘activated’ by inflammatory stimulus, which is required for efficient remyelination and is thought to confine OPC activity to injury sites and away from normal CNS white matter [11,12]. Upon reaching the lesion site, OPCs then begin the process of differentiation into oligodendrocytes and the ensheathment of axons with fresh myelin [13,14]. At the same time, myelin debris that has been produced as a consequence of demyelination must be cleared from the lesion site by phagocytes, as free myelin inhibits efficient remyelination [15]. Each step in the differentiation process is associated with expression of characteristic oligodendroglia transcription factors, lipids and surface proteins. In research, these can serve as markers for OPC development into mature oligodendrocytes. They include the pan-oligodendroglia markers oligodendrocyte transcription factor 2 (OLIG2) and SRY-box transcription factor 10 (SOX10), proliferative or immature OPC markers like platelet-derived growth factor receptor alpha (PDGFRα) and neural/glial antigen 2 (NG2), differentiation markers like galactocerebrosidase (GalC) and oligodendrocyte marker 4 (O4), and mature markers associated with the myelin sheath like myelin basic protein (MBP), myelin proteolipid protein (PLP) and myelin-associated glycoprotein (MAG) [16].

Other glial cells mediate and support the remyelination process, and the specific roles of microglia and astrocytes have both recently been reviewed [17,18]. In brief, during MS and other neuroinflammatory diseases, microglia become polarized towards a pro-inflammatory or ‘M1-like’ phenotype. These pro-inflammatory microglia secrete inflammatory cytokines, promote BBB permeabilisation, recruit and mediate differentiation of peripheral immune cells like T cells into the CNS, present antigens and phagocytose debris. As such, M1-like microglia have a clear and central role in MS pathology [19]. However, microglia can also be polarized, by cytokines like interleukin 4 (IL-4), IL-10 and IL-13 towards an anti-inflammatory or ‘M2-like’ phenotype. This anti-inflammatory polarisation state is associated with resolution of CNS damage and neurogenesis, and furthermore has been specifically implicated in mediating OPC differentiation and remyelination [20]. Interestingly, recent evidence indicates that the pro-remyelination activity of microglia requires necroptosis and depletion of pro-inflammatory microglia and subsequent expansion or ‘rebirth’ of anti-inflammatory microglia, challenging previous assumptions that a simpler process of polarising and re-polarising a more constant population of microglia was involved [21]. Taken with the essential task of phagocytosing myelin debris, microglia have a substantial role in supporting remyelination. Astrocytes, on the other hand, are activated during CNS injury in a process known as reactive astrogliosis. During reactive astrogliosis, astrocytes may proliferate, respond to and secrete inflammatory mediators like cytokines, produce neuroprotective agents in response to oxidative stress like glutathione, modify and/or repair BBB and can form glial scar tissue in the CNS [22]. In MS, astrocytes play a number of essential roles in remyelination, such as iron efflux that supports OPC differentiation [23], cholesterol transfer to oligodendrocytes, production of regenerative factors like osteopontin, forming gap junctions with oligodendrocytes that is essential for myelin maintenance and survival [24], cross-talk with microglia [25] and glial scar formation [26].

Experimentally, there has been an increase in the capacity to understand remyelination processes using in vivo and ex vivo animal models, along with patient samples. An in vivo disease model frequently used is the experimental autoimmune encephalomyelitis (EAE) demyelinating autoimmune disorder. While not without important limitations with respect to modelling MS, the EAE model does induce substantial peripheral immune invasion of the CNS, particularly the spinal cord, and T cell-mediated destruction of myelin that leads to deficits in motor function [27]. Two other notable in vivo models used in MS studies are the lysolecithin (LPC) and cuprizone toxin-induced models of demyelination [28]. A key limitation of these models is that they do not recapitulate the autoimmune-mediated damage of MS. However, both induce the characteristic destruction of myelin sheaths, and provide approaches that specifically study the mechanisms behind remyelination that is key in developing regenerative therapies for MS [29].

In vitro and ex vivo models that are useful in studying remyelination include murine primary oligodendrocyte cultures and organotypic brain slice cultures. Murine primary oligodendrocyte cultures can both be used to examine the process of oligodendrocyte differentiation, and as such have utility in high-throughput drug screening in MS drug discovery. A clear limitation of these approaches is that the cells are out of structure, so important intercellular interactions may be lost. Ex vivo murine organotypic brain slice cultures can be used to address this limitation, as they feature all major cell types of the CNS and retain cellular organisation. Demyelination can be experimentally induced in these cultures using LPC or immune-mediated insults, making them a natural complement to and useful midpoint between in vitro and in vivo models described above. Compared with other in vitro models, however, slice cultures are lower throughput and more laborious, and also lack the influence of the peripheral immune system. Taken together though, in vitro and models of remyelination are a useful tool for preliminary research into regenerative therapies in MS [30].

Turning to patient samples, one approach is dissection of brain and spinal cord tissue from deceased patients. Modern techniques of microdissection can even allow for single cells to be isolated and examined, which allows for high spatial resolution examination of lesions and study of particular cell types of interest [31]. A further advantage of this approach is that the nature of MS is that patient samples may be identified that have lesions in different stages of resolution—active, inactive and resolved at time of death—along with healthy grey and white matter than can serve as appropriate control samples, minimising variation [32]. An important limitation is that most MS patients will have been on one or more disease-modifying therapies (DMTs) for several years or decades prior to death. Several DMTs alter the number and character of CNS lesions in MS [33], so any observations made could be attributable to drug effects and side-effects rather than the disease itself. Available samples may also tend to be skewed towards older patients, and temporal resolution is necessarily limited to a single point in time. Studying remyelination in living patients is more challenging. An approach using lesion magnetisation transfer ratios in MRI scanning has been described [34], but this has the clear drawback of not allowing for molecular examination of remyelinating lesions, and instead the main utility is in assessing efficacy of remyelinating agents. Induced pluripotent stem cells (iPSCs) taken from MS patients are an emerging strategy to more directly study remyelination using patient samples. MS iPSCs can be used to generate oligodendrocytes and repeat many of the experiments described above that rely on murine oligodendrocytes—this could have particular future utility in developing personalized treatments for individual MS patients [30]. iPSCs have also been recently used to develop three-dimensional neural cultures that include differentiation of OPCs and subsequent myelination of cultured neurons. This technique could also see significant utility in future studies to more accurately replicate the conditions of MS damage in human cell cultures [35].

### 1.3. MicroRNAs

MicroRNAs (miRNAs) are small (~21–24 nucleotide) non-coding RNA molecules that are known to modulate a wide variety of cellular processes, with each miRNA capable of regulating the post-translation expression of a substantial number of genes. The general scheme of miRNA biogenesis is well understood and has been thoroughly reviewed [36]. Broadly, miRNAs are generated by RNA Polymerase II as a primary transcript (pri-miRNA), then subsequently processed by the microprocessor complex, consisting of the enzymes Drosha and DGCR8, into a ~70 nucleotide hairpin loop. This precursor miRNA (pre-miRNA) is then exported to the cytoplasm by the activity of exportin 5, where the enzyme Dicer cleaves it into a double-stranded miRNA duplex. One of these strands—the guide strand—is then bound to Argonaute and used to form an RNA-induced silencing complex (RISC) that will guide the miRNA sequence to target mRNAs, where it serves as a binding template. In most cases, a 6–8 nucleotide sequence (seed sequence) at the 5′ end of the miRNA will bind to the 3′ untranslated region (UTR) of one of perhaps several hundred mRNA targets, at which point the mRNA is degraded and translation inhibited. However, alternative mechanisms have also been observed, including miRNAs that can induce or enhance mRNA and protein expression rather than inhibiting it [37,38].

MiRNAs are central regulators of both the immune and central nervous systems, and so have been a major topic of research in autoimmune and neurological disorders, including MS [39,40]. The potential for miRNAs as biomarkers for MS is one area that has received considerable interest, and several studies have identified groups of miRNAs in that can distinguish between RRMS, SPMS and PPMS using plasma, serum, circulating exosome, cerebrospinal fluid (CSF) or peripheral blood mononuclear cell (PBMC) samples [41,42,43,44,45,46]. The expression of some miRNAs correlates with the progression index of patients [47], and miRNAs can also identify relapsing and remitting phases of the disease [46] or serve as biomarkers for response to particular treatments [48]. miRNAs have also been identified as having important roles in multiple aspects of MS, for example miR-155 which is involved in activation of both T cells and macrophages, the permeability of the BBB, and neurodegeneration following immune-mediated destruction of the myelin sheath [49]. However, the role of miRNAs in the remyelination process has not been as well characterised, but is clearly important to the process.

The importance of miRNAs generally in remyelination is underscored by knockdown models of Dicer, a critical enzyme in the miRNA maturation process. Oligodendroglia-specific Dicer knockdown in mice inhibits normal developmental myelination, and when cultured in vitro these OPCs do not differentiate past the proliferation stage [50,51]. The knockdown also leads to oxidative damage, inflammatory astrocytosis and microgliosis in the brain, and eventually neuronal degeneration and shorter lifespan [52]. In MS patients, Dicer is underexpressed in B cells and associated with increased expression of CD80, and this mechanism potentially contributes to the activation of the abnormal MS immune response [53]. Furthermore, in the EAE model of MS, the assembly of the RISC complex that is essential for bioavailability of miRNAs is significantly disrupted; in particular, the constituent proteins Ago2 and FXR1 are downregulated in oligodendrocytes and infiltrating T cells, leading to aberrant miRNA expression and activity [54]. 

In this review, we describe the specific role of miRNAs in remyelination in each major cell type involved in the process. We then consider the utility of miRNAs in treatments aimed at restoring remyelination, and the challenges and opportunities associated with different potential strategies. 

## 2. Contribution of miRNAs to Remyelination in CNS Cells

**Figure 1:**
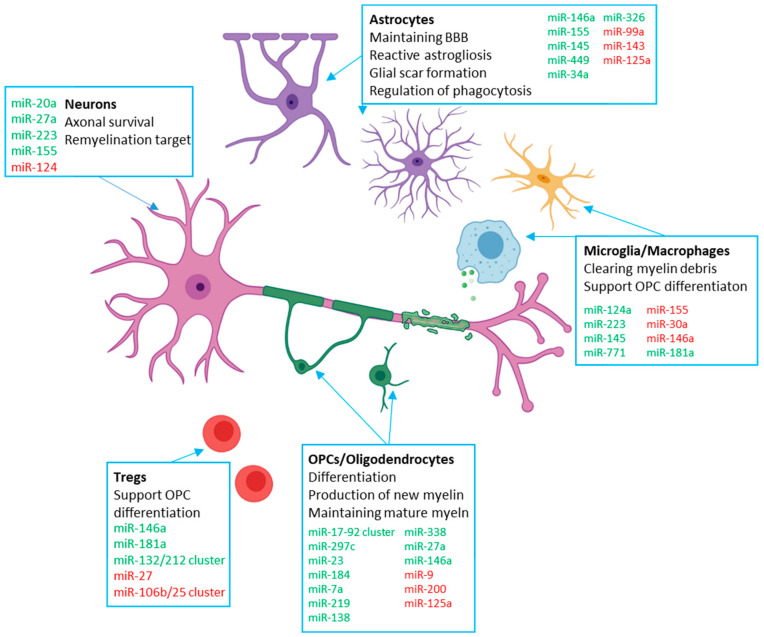
Schematic of key cells involved in remyelination and relevant miRNAs that regulate the process. miRNAs and miRNA clusters that are broadly positive regulators of remyelination are listed in green, and those that are broadly negative regulators of remyelination are listed in red (created with BioRender.com).

In this section, we take each major cell types involved in remyelination and examine the contribution of miRNAs to the process. A summary of these roles is provided in Table 1, and a schematic provided in Figure 1.

### 2.1. OPC Intrinsic miRNAs

Oligodendrocytes are the myelin-producing cells of the CNS, and repairing demyelinated lesions in MS critically rests on the recruitment of fresh OPCs into the damaged region and their successful differentiation into mature, myelinating oligodendrocytes. This process of differentiation is tightly regulated by several factors, including transcriptional control, exogenous signalling from molecules like cytokines, chemokines and neurotransmitters [82,90], as well as epigenetic mechanisms that have been reviewed recently [91]. Here, we specifically focus on the contribution of microRNAs in this process.

It is important first to note that just as remyelination occurs in a different context to developmental myelination, the miRNA expression profile of adult OPCs is substantially different to that of both mature oligodendrocytes and fetal OPCs, indicating differential regulation of these two processes. In adult OPCs, miR-449a, -145, -483, -22, -338, -490, -184, -100, -181a, -99a-, and -214 show low expression compared with relatively high expression different fetal OPC subtypes, while miR-219 (especially miR-219-5p) and miR-155 are more highly expressed in adult OPCs compared with fetal groups [92]. With this in mind, there are several oligodendrocyte intrinsic miRNAs and miRNA clusters that contribute to the process of remyelination. 

Three key OPC miRNAs in supporting remyelination are miR-219, miR-138 and miR-338, and are the most highly induced miRNAs during differentiation [50]. In DICER knockdown cells associated with failure of normal myelination, transfection of miR-219 could partially rescue defective OPC differentiation, and in normal OPCs overexpression of miR-219 alone or with miR-138 is sufficient to promote differentiation [50,51]. Interestingly, miR-219 appears to be important in both early and late stages of OPC differentiation, while miR-138 promotes initial differentiation while delaying later differentiation, indicating a miRNA-mediated mechanism of temporal control of full differentiation [50]. Furthermore, miR-219 is important in the maintenance of healthy myelin sheath. Postnatal knockdown of DICER results in excess lipid accumulation in myelin-rich brain regions, inversely correlated with miR-219 and correlated with the miR-219 target ELOVL7 [52]. Deletion of miR-219 impairs both developmental myelination and remyelination, an effect exacerbated by co-deletion of miR-338 [56]. In the cuprizone model, miR-219 overexpression is associated with reduced demyelination [55], and in both EAE and the LPC model of demyelination, delivery of miR-219 enhances remyelination [56]. miR-219 and miR-338 are also both downregulated in white matter lesions of PPMS patients [57].

Several other OPC miRNAs positively or negatively regulate remyelination. On the side of broadly positive regulation of remyelination, miR-146a overexpression in OPCs promotes differentiation and remyelination in the cuprizone model [62], although deletion of this miRNA is also associated with a relative increase in 2′3′-cyclic nucleotide 3′-phosphodiesterase(CNP)+ oligodendroctyes during demyelination and a decrease in NG2+ OPCs, pointing to a complex role in OPC differentiation and remyelination [93]. The miR-17-92 cluster enhances OPC proliferation in vitro and deletion reduces oligodendrocyte numbers in vivo, indicating a role in OPC expansion [67]. miRNAs in the *Sfmbt2* cluster fall in oligodendrocytes 6 weeks post-treatment in the cuprizone model before returning to control levels during recovery; the cluster member miR-297c in particular promotes G0/G1 cell cycle arrest and differentiation in OPCs [68]. miR-23 ensures full maturation of oligodendrocytes and contributes to myelin maintenance by inhibiting lamin B1 [71]. Prior to proliferation and differentiation, miR-184 in neural progenitor cells (NPCs) commits them to an oligodendrocyte lineage by suppressing positive regulators of neuron and astrocyte differentiation, while also enhancing myelination [72]. miR-7a is highly enriched in OPCs and overexpression in NPCs and the embryonic mouse cortex promotes generation of oligodendrocyte lineage cells, while also halting further maturation [73].

On the side of broadly negative regulation of remyelination, miR-125a impairs OPC differentiation in vitro and in the LPC model while silencing enhances in vitro differentiation and remyelination following LPC demyelination, and furthermore is upregulated in the CSF of RRMS patients with actively demyelinating lesions [94,95]. miR-27a is increased during specification of embryonic stem cells into OPCs [96], is expressed throughout oligodendrocyte development, and loss of miR-27a is associated with reduced levels of mature oligodendrocytes. However, overexpression inhibits both OPC proliferation and differentiation and impairs remyelination in the cuprizone model, and miR-27a is also overexpressed in MS lesions, indicating that a steady-state level of expression is required for efficient remyelination [60]. Overexpression of miR-9 and miR-200 impairs OPC differentiation [69], and miR-9 in particular is downregulated during OPC differentiation and appears to suppress the translation of the peripheral myelin protein PMP22 in the CNS [70]. 

### 2.2. Microglia

Microglia are macrophage-like cells distributed heterogeneously across the brain and spinal cord, and form the resident innate immune system of the CNS. The primary role of microglia is to survey the CNS and respond to infection or damage; this is accomplished via pathogen-associated molecular pattern (PAMP) and damage-associated molecular patter (DAMP) receptors, respectively [97]. However, they also play clear roles in the remyelination process of which a number of miRNAs have been described to have a role. MiR-146a is generally associated with tempering pro-inflammatory microglia polarisation [63], and loss of miR-146a was associated with reduced microglia numbers and both reduced demyelination and stunted remyelination in the cuprizone model [93], pointing to a multi-faceted role of both microglia and this miRNA. Exogenous miR-146a could increase anti-inflammatory activation of microglia, an effect associated with increased OPC differentiation and remyelination [64]. miR-124 promotes a quiescent, steady-state phenotype and suppresses EAE; it is downregulated in activated microglia in EAE [74] and in both pro-inflammatory and anti-inflammatory polarized microglia in vitro [84]. Following lysolecithin-induced demyelination, miR-223 is required for efficient M2-like activation of microglia associated with phagocytosis of myelin debris and supporting remyelination [76]. However, miR-223 deficiency reduces CNS demyelination, delays disease onset and ameliorates neuroinflammation in EAE, indicating a complex or finely-balanced role for miR-223 in remyelination [77]. Overexpression of miR-30a in microglia leads to the secretion of factors that promote apoptosis in OPCs, inhibiting their differentiation [83]. MiR-155 is upregulated in pro-inflammatory microglia—by one measure, the most upregulated miRNA [84]—and is also highly expressed in pro-inflammatory microglia in MS. Inhibition of miR-155 reduced inflammatory cytokine production of microglia [79], and pro-inflammatory polarized microglia expressing higher levels of miR-155 appear to be less efficient at myelin phagocytosis [78]. MiR-145 and miR-771 are also strongly associated with the anti-inflammatory activation of microglia that supports remyelination [84,98]. However, a direct connection has yet to be demonstrated.

### 2.3. Astrocytes

Astrocytes are the most abundant glial cells in the CNS, and are widespread across both the grey and white matter of the brain. Astrocytes have a number of important functions in the healthy CNS, such as providing energy and metabolic support to neurons, supporting synaptic transmission, and helping to form, maintain and regulate the BBB [99].

Astrocyte miRNAs can be both supportive and inhibitory of pro-remyelination processes. In microdissections of MS lesions, miR-99a, miR-143 and miR-145 are downregulated in astrocytes across white matter lesions and in active grey matter lesions, while miR-449 and to a lesser extent miR-125a are upregulated in active white matter lesions in patients samples [32]. These miRNAs are all regulators of glial scar formation, which first requires the initiation of reactive astrogliosis and subsequent astrocyte proliferation at the lesion site. The scar then matures—at which point, proliferation and reactive astrogliosis are tempered back down [100]. Decreased miR-99a/143 boosts local astrocyte proliferation and miR-145 inhibits astrogliosis, miR-449 attenuates scar formation, and miR-125a promotes astrogliosis [32]. The glial scar has a complex role in efficient remyelination—it can limit OPC migration and therefore inhibit repair, but also restricts inflammation to the lesion site and prevents damage to the wider parenchyma [99]. Furthermore, glial scar astrocytes can release pro-differentiation factors for OPCs and do not prohibit effective remyelination in rat EAE [101]. These miRNAs may therefore be important to maintaining a desirable balance for lesion resolution. 

Astrocyte miR-155 and miR-146a were downregulated in active lesions, suggesting that astrocytes withdraw from inflammatory activation during lesion formation [32]. These miRNAs are also both reduced in astrocytes following treatment with the MS therapy dimethyl fumerate (DMF), reducing the inflammatory response to IL-1β [65]. However, miR-155 is upregulated in astrocytes in phagocytically active MS lesions and reduces expression of CD47, an inhibitor of microglia/macrophage phagocytosis. This downregulation releases phagocytes to clear myelin debris, an essential step in remyelination, and may also be mediated by miR-34a and miR-326 [80]. It may therefore be the case that astrocyte upregulation of miR-155 helps resolve MS lesions, and that the lack of this miRNA in some active lesions is indicative of dysfunctional clearance of myelin. Finally, miR-125a was diminished at the BBB of MS patients, which is associated with reduced barrier formation capacity in astrocytes [58]. Individual miRNAs can therefore have an impact on several astrocyte processes that are relevant to remyelination.

### 2.4. Neurons

Neurons are the most important component of functioning nervous tissue, and are the electrically excitable cells that are primarily responsible for the transmission and organisation of information that aggregates into the highly diverse and specialized functions of the nervous system. The characteristic demyelination that occurs in MS leads to disease symptoms because of the disruption this causes to neuronal function—demyelinated axons are less efficient at transmitting electrical signals, and also become vulnerable to more severe damage and even cell death [102,103]. As such, preservation of axons is required for remyelination to occur, and the neurons themselves also provide signals to surrounding glia that regulate and promote resolution of lesions and myelin repair [104].

A number of neuronal miRNAs have been implicated in axon survival and remyelination in MS. MiR-124 is upregulated in demyelinated hippocampal neurons in patient autopsies, and upregulation was associated with lower expression of the ionotropic receptors AMPA 2 and AMPA 3. This upregulation is recapitulated in demyelinated mouse hippocampal neurons and is associated with memory dysfunction, while remyelination reversed these changes [75]. MiR-223—which also has a role in microglia and macrophages in remyelination—and miR-27a are upregulated in retinal ganglion cells in EAE following local damage, and are associated with neuroprotection from glutamate toxicity [61]. In the spinal ventral horn during EAE, Vitamin D Receptor (VDR) expression on neurons is reduced, while miR-125a is upregulated, and both activation of the VDR and inhibition of miR-125a are associated with reduced clinical scores, suggesting a neuroprotective role [59]. In the cuprizone model, miR-155 and miR-20a upregulation during demyelination may also be important for neuronal survival and remyelination by inducing the Nogo Receptor via suppression of mothers against decapentaplegic homolog 2 (SMAD2) and SMAD4. However, this study was limited to whole-cerebrum miRNA expression [81]. Finally, in EAE, several miRNAs—miR-7a, miR-101a, miR-142a, miR-199b, miR-203, miR-205, miR-340, miR-370, miR-374b, miR-381, miR-1969 and miR-7056—are upregulated in retinal ganglion cells and *in silico* analysis suggests that they may impair neuroprotective pathways, which in turn may present an obstacle to efficient remyelination [105]. 

## 3. Peripheral Immune miRNAs

Moving beyond the resident cells of the CNS, the cells of the peripheral immune system are well established to have a critical role in the pathology of multiple sclerosis, and indeed most approved treatments for the disease target the peripheral immune system in some way to exert their primary therapeutic effect [106]. While MS has classically been considered to primarily be a T cell-mediated disorder, recent evidence suggests that all cell types of the peripheral immune system can be implicated in susceptibility to the disease [107]. In terms of remyelination, specifically, several peripheral immune cell types have been implicated in supporting or otherwise influencing the process, and miRNAs may have a role in the activity of these cells during MS.

### 3.1. Leukocytes

T cells are a central component of the adaptive immune system, and are well established to play an important role in MS pathogenesis. T cells can be broadly categorized into two lineages—helper T cells which are defined by expression of CD4, and killer T cells defined by expression of CD8. Both CD4^+^ and CD8^+^ T cells are implicated in MS-related autoimmunity and are considered major effectors of the disease. Naïve CD4^+^ T cells differentiate into one of several distinct phenotypes that have diverse immunological functions. In MS, the activity of Th1 and Th17 T cells are associated with demyelination and disease progression. However, recent work has found that Tregs may play an important role in supporting remyelination [108,109]. In EAE, Treg-deficient mice show impaired remyelination and OPC differentiation in spite of similar lesion sizes and demyelination burden, suggesting a remyelination-specific role for these cells [110]. Treg-secreted factors also support remyelination and OPC differentiation in a variety of ex vivo and in vitro models [110], and furthermore Tregs are required for CNS repair in models of spinal cord injury (SCI) [111].

Several miRNAs have been implicated in Treg differentiation and activity in MS and other autoimmune disorders. miR-181a, which is downregulated in the white matter of MS patients and in the spinal cord of acute and chronic EAE mice, promotes Treg differentiation alongside inhibiting Th1 differentiation and pro-inflammatory macrophage polarisation [85]. miR-146a, which as detailed above is involved in regulating CNS cells during remyelination, additionally blocks Th17 differentiation via IL-6 and IL-17 in CD4^+^ T cells during EAE, and deficiency in the miRNA inhibited Treg differentiation [66]. Excess expression of miR-27 impairs both Treg differentiation and Treg immune suppressive activity [86]. The miR-132/212 cluster is important for the differentiation of Th1 and Th17 T cells, and suppression of this cluster in EAE reduced differentiation of these subtypes and ameliorated disease progression while not interfering with Treg cell numbers [87]. In contrast, the miR-106b/25 cluster is upregulated in Tregs from MS patients—the authors speculate that dysregulation of this cluster impairs Treg suppressor function by interfering with the TGF-β pathway [88]. Finally, myelin-specific CD49d^+^CD154^+^ lymphocytes can impair OPC differentiation via inducing excessive miR-665, resulting in immune-reactive oligodendrocytes with dysfunctional miRNA and myelin synthesis that could be reversed by suppressing miR-665 in the affected oligodendrocytes [60].

### 3.2. Monocytes

Monocyte-derived macrophages are another major cell type of the peripheral immune system that have a major contribution to pathology in MS. One difficulty in studying the impact these cells have, however, is the difficulty in distinguishing infiltrating peripheral macrophages from CNS-resident microglia in and around MS-like lesions. As such, many older studies investigating the contribution of infiltrating macrophages and resident microglia in MS either used controversial approaches to classify cells into one or the other [112], or simply did not attempt to distinguish the cell types. Recent innovations in both methodology and scientific understanding, however, have allowed for separate classification of these cells with greater precision and accuracy, and it has now been established that infiltrating peripheral macrophages have a specific and important role in resolution of demyelinating lesions in EAE [113]. Establishing the specific miRNAs that are involved in pro-remyelination peripheral macrophage responses will require application of these techniques to distinguish them from microglia miRNAs. Some pro-remyelination microglia miRNAs like miR-223, however, have already been shown to play a similar function in peripheral macrophages, suggesting that there may be considerable overlap even after precise subtyping of these macrophages in MS [76].

## 4. miRNAs as Therapeutic Targets in Multiple Sclerosis

Given the clear and substantial role that they have in several aspects of the remyelination process, the potential for miRNAs as therapeutic targets or as therapeutic agents themselves as a strategy to develop novel treatments for supporting repair and remyelination in MS has received considerable interest in recent years. Core strategies in miRNA therapeutics include synthetic mimics that replicate the activity of miRNAs [114], miRNA inhibitors or ‘antagomIRs’ that bind to and block the activity of endogenous miRNAs [115], long non-coding RNAs (lncRNAs) that act as ‘sponges’ for miRNAs and reducing their effect on target mRNAs [116], target-site blockers (TSBs) that are specific to the site of miRNA binding on a particular mRNA and compete for binding, reducing miRNA regulation of a specific mRNA [117], and other pharmacological treatments that regulate the expression of individual miRNA-coding genes and miRNA clusters [118].

One challenge that is common to miRNA therapeutics in practically all disease contexts is the potential for undesirable off-target effects. miRNAs, including those described above, typically have hundreds of target genes, therefore significantly modifying the activity of even a single miRNA can result in the expression of several genes that are unrelated to the desired therapeutic effect being concurrently altered. Indeed, in remyelination the same miRNA can have both beneficial and detrimental effects in different contexts. Cell-specific effects of miRNAs should also be considered when approaching therapeutics. miR-146a mimics can promote differentiation of OPCs into myelinating oligodendrocytes along with M2-like activation of microglia and macrophages that is associated with repair [64]. Similarly, infusion of miR-146a mimics into the corpus callosum of mice undergoing the cuprizone model supported OPC differentiation and synthesis of myelin basic proteins [62]. However, when transferred to hippocampal neurons via microglia EVs, miR-146a damages dendritic spine density by reducing expression of presynaptic SYT1 and postsynaptic NLG1, an effect not observed when EVs were obtained from pro-repair M2-like microglia or when microglia were pre-treated with a miR-146a antagomiR [119]. MiR-146a knockout mice have reduced OPC counts and remyelination capacity in the recovery phase of the cuprizone model, but also have reduced inflammation, axonal damage, demyelination and macrophage infiltration during the demyelinating phase, further indicating a disease stage-specific effect of this miRNA [93]. Treatment strategies involving miRNAs to promote remyelination should therefore consider both the appropriate disease stage and cell-specific targeting to minimize undesirable side-effects and maximize therapeutic potential. 

A challenge common to all treatments of CNS diseases is ensuring that the therapeutic agent can cross the BBB, and MS is no exception. This is particularly important in developing treatments for the progressive forms of MS, as the BBB is less porous than in RRMS and most of the damage is thought to occur ‘behind’ the BBB and infiltrating peripheral immune cells play a more limited role in this stage of the disease [5]. In the context of miRNA therapeutics, several approaches can be taken to address this difficulty. miRNA mimics that have been modified to resist degeneration can cross the BBB following systemic administration, at least in the context of active EAE, where miR-146a mimics accumulate in the CNS, improving neurological function and enhancing remyelination [64]. Intranasal administration of mimics can also achieve non-invasive delivery to CNS cells, such as in delivering miR-146a mimics to the hippocampus of mice in an Alzheimer’s disease model [120]. Synthetic nanoparticles, such as PLGA nanoparticles, can be engineered to carry miRNA mimics or antagoMIRs, and given appropriate size and modifications can cross the BBB [121]. 

However, a more promising particle-based strategy may be using extracellular vesicles (EVs) [122]. EVs are membrane-bound particles, typically 30–1000 nm in diameter, of cellular origin and can be further subcategorized into exosomes, microvesicles and apoptotic bodies based on size and mechanism of biosynthesis [123]. EVs are important mediators of intracellular signalling, transferring their cargo which may consist of proteins, lipids and genetic material like miRNAs between cells [124]. EVs present several advantages over nanoparticles for delivery of CNS-bound therapeutics. They readily cross the BBB with low immunoreactivity, see reduced clearance by the mononuclear phagocyte system compared with synthetic nanoparticles, and can be selected or bioengineered to be preferentially taken up by particular cell types, such as via modification of the surface protein profile [125]. Indeed, EVs isolated from HEK293 cells engineered via lentiviral infection to express miR-219a, cross the BBB, promote OPC differentiation and improve clinical score in EAE, outperforming both PLGA nanoparticles and DSPC liposomes carrying miR-219a mimics [126]. Exosomes taken from dendritic cells pre-treated with IFNγ promote ex vivo remyelination and in vivo myelination compared with untreated control exosomes, an effect associated with increased exosomal miR-219 cargo [127]. Exosomes taken from the serum of both young rats and rats kept in an enriched environment had increased levels of miR-219, which promoted OPC differentiation in vitro and enhanced in vivo myelination when intranasally administered to ageing rats [128]. Looking at specific cell sources, exosomes taken from PBMCs, T cells, B cells and dendritic cells of environmentally-enriched mice could all promote remyelination ex vivo, and variously had increased levels of miR-219, -181, -9, -17 and -665 [129]. Aside from exogenous EV enrichment and administration, cells that secrete exosomes with pro-remyelination miRNA cargo could be directly transfected into a patient, as has been demonstrated in vivo with macrophages modified to synthesize and secrete the enzyme catalase in a Parkinson’s disease model [130]. Exosome cargo can also be modified following enrichment from cell sources, using methods like electroporation, lipofection and sonication to directly load exogenous miRNA prior to administration [131]. Taken together, a variety of strategies are available to generate exosomes enriched in pro-remyelination miRNAs, and preferentially target particular cell types of the CNS upon administration. 

A strategy to circumvent the BBB entirely is directly implanting biomaterial constructs engineered to secrete and deliver particular miRNAs to the cells of the CNS. This approach has mainly been studied in models of spinal cord injury (SCI). However, the nerve repair process also involves remyelination of axons via OPC differentiation to mature oligodendrocytes [132], and so may have relevance for remyelination in MS lesions. Recent work has found that poly(ɛ-caprolactone) (PCL) nanofibers loaded with both miR-219 alone and miR-219/miR-338 mimics can efficiently deliver these miRNAs to OPC cell cultures, promoting their differentiation into mature, myelin-producing oligodendrocytes [133]. This approach was particularly effective when the fibres mediating delivery of the miRNAs were aligned and small, indicating topographic effects on the efficiency of treatment [134]. Moving to in vivo applications, modified poly(caprolactone-co-ethyl ethylene phosphate) (PCLEEP)-aligned fibres and collagen hydrogel scaffolds loaded with miR-219/miR-338 and neurotrophin-3 preserved more oligodendrocyte lineage cells following SCI in mice. These scaffolds also increased the rate and extent of OPC differentiation into mature oligodendrocytes, and at the site of implantation greater myelination of axons, myelin density and MBP concentration was observed [135]. Scaffolds of this configuration also promoted OPC differentiation and remyelination in rat SCI, and furthermore reduced pro-inflammatory cytokine secretion from microglia and inhibited reactive astrocyte activation [136]. Combining stem cell and biomaterial approaches, implantation of human endometrial stem cells encapsulated in a fibrin hydrogel scaffold is associated with improved motor recovery in SCI, and when the stem cells were further engineered to overexpress miR-219 remyelination also improved [137]. Finally, in a peripheral model of sciatic nerve crush, a biodegradable and biocompatible cationic polymer generated from polyethyleneimine cross-linked with 2,6-pyridinedicarboxaldehyde was loaded with miR-221/miR-222 and used to transfect Schwann cells, promoting MBP synthesis and remyelination [138].

Gene therapy via lentiviral vectors has seen considerable research being carried out in clinical trials in recent years [139], and there are several possible strategies for their use in modifying miRNA activity to promote remyelination. Following LPC-induced demyelination in vivo, lentiviral-mediated inhibition of miR-125a promoted remyelination by enhancing OPC maturation, while conversely overexpression of miR-125a by the same means inhibited OPC differentiation and subsequent myelin repair. Furthermore, miR-125a is upregulated in the white matter of active lesions, suggesting that this approach could normalize a defective repair mechanism in MS [94]. Taurine-upregulated gene 1 (TUG1) is an lncRNA that acts as a sponge for miR-9; lentiviral downregulation of TUG1 in EAE improved disease scores and reduced inflammation [140]. However, as described above, miR-9 is also downregulated in OPC differentiation, so this benefit may not be based on enhanced repair. A final, interesting strategy used lentiviral-mediated forced expression of the miR-302/367 along with valproate treatment in the cuprizone model, which converted astrocytes into myelinating oligodendrocytes, enhancing remyelination and improving behavioural impairments [141].

Stem cell therapies to promote remyelination can also be enhanced by miRNA modification. Embryonic stem cells overexpressing miR-219 rapidly differentiate into oligodendrocyte lineage cells; transplanting these cells into mice in the cuprizone model of demyelination not only directly promotes remyelination and improved cognitive function by supplying fresh OPCs, but also enhances the proliferation of host endogenous NPCs following chronic demyelination [142].

Finally, there are treatment strategies that may not directly target mature miRNAs but may still alter miRNA expression and activity alongside other therapeutic roles. Fingolimod, an approved treatment for relapse-remitting multiple sclerosis, is thought to primarily exert disease-modifying effects via trapping lymphocytes in lymphoid organs, preventing migration to the CNS [143]. However, fingolimod has also been shown to have pro-remyelination effects following LPC-induced demyelination [144,145] and, within 5 h of initial administration to patients, substantially changes the miRNA profile of serum EVs, including several implicated in remyelination such as downregulation of miR-223 and upregulation of miR-155 [146]. In patients that respond to treatment with interferon, platelet expression of miR-26a is upregulated and the target gene *SLC1A1* is conversely downregulated, a relationship that was first validated in OPCs and implicates the glutamate receptor signalling pathway that is altered in MS [147]. In an RRMS patient cohort with aberrant expression of miR-326, miR-155, miR-146a and miR-142-3p compared to controls, glatiramer acetate treatment reduced miR-146a and miR-142-3p, in contrast to interferon-beta, which had no impact on these miRNAs [148]. In both patient white matter lesions and EAE, miR-155 and miR-338 were upregulated and associated with inhibition of neurosteroid synthesis, particularly allopregnanolone. Treatment with allopregnanolone could then improve EAE scores and myelin damage [149]. Following successful autologous hematopoietic stem cell transplantation, miR-155, miR-16 and miR-142 see sustained downregulation in CD4^+^ and CD8^+^ T cells of MS patients, along with an increase in the number of Treg cells that are associated with myelin repair [53]. Sulfasalazine inhibits M1-like microglia activation in the cuprizone model, both by increasing levels of miR-136, which targets AKT2-NFκB and by decreasing lnc HOTAIR expression, which normally sponges miR-136, and treatment is overall associated with greater remyelination [150]. Finally, an interesting recent in vitro study applied low-frequency pulsed electromagnetic field (PEMF) stimulation to cultured OPCs, which promoted the differentiation of OPCs by upregulating the expression level of miR-219 and downregulating the expression level of Lingo1 [151]. PEMF is a potential adjunct therapy in SCI [152], which, as mentioned above, involves remyelination, so this pro-OPC differentiation effect via miR-219 may also be of interest in MS.

## 5. Conclusions

This review has discussed the substantial and diverse role that miRNAs have in the central repair process in MS, remyelination. The effect of miRNAs is dynamic and not limited to any particular cell type, and indeed a complex interplay of several miRNAs across numerous different cells found in the CNS during MS can be described at each stage of the process. For this reason, miRNAs are a promising avenue of research in developing novel therapeutics that aim to restore remyelination in MS, and the application of cutting-edge techniques in modern medicine like nanomedicine, biomaterials, gene therapy and stem cell implants can allow the potential of miRNAs to be fully exploited in treating both MS and other diseases of the CNS.

## Figures and Tables

**Table 1:** Summary of miRNAs involved in remyelination.

miRNA	Cell Type	Role in Remyelination
**miR-219**	Oligodendrocytes	- Promotes early and late stages of OPC differentiation [50,51,55,56] - Maintenance of mature myelin sheath [52]
**miR-138**	Oligodendrocytes	- Promotes early and inhibits late OPC differentiation [50]
**miR-338**	Oligodendrocytes	- Supports miR-219 in promoting OPC differentiation [56,57]
**miR-125a**	Oligodendrocytes Astrocytes Neurons	- Impairs OPC differentiation - Promotes astrogliosis [32] - Supports BBB maintenance [58] - Reduction is neuroprotective [59]
**miR-27a**	Oligodendrocytes Neurons	- Steady-state expression needed for OPC specification, proliferation and differentiation [60] - Increase is neuroprotective [61]
**miR-146a**	Oligodendrocytes Microglia Astrocytes T cells	- Promotes OPC differentiation [62] -Tempers pro-inflammatory microglia activation and promotes anti-inflammatory activation [63,64] - Reduction associated with astrocyte withdrawal from inflammatory activation [32,65] - Inhibits Th17 differentiation while supporting Treg differentiation [66]
**miR-17-92 cluster**	Oligodendrocytes	- Promotes OPC proliferation [67]
**miR-297c**	Oligodendrocytes	- Promotes G0/1 cell cycle arrest and OPC differentiation [68]
**miR-9**	Oligodendrocytes	- Overexpression impairs differentiation [69] - Suppresses expression of peripheral myelin protein [70]
**miR-200**	Oligodendrocytes	- Overexpression impairs differentiation [69]
**miR-23**	Oligodendrocytes	- Inhibits lamin B1, supporting myelin maintenance [71]
**miR-184**	Oligodendrocytes	- Commits NPCs to OPC lineage, expression enhances myelination [72]
**miR-7a**	Oligodendrocytes	- Promotes generation of OPCs, inhibits maturation [73]
**miR-124**	Microglia Neurons	- Promotes quiescent state [74] - Upregulation in demyelinated hippocampal axons associated with memory dysfunction [74,75]
**miR-223**	Microglia Neurons Macrophages	- Required for efficient anti-inflammatory activation and phagocytosis of myelin debris [76] - Increase is neuroprotective [61] - Overall deficiency ameliorates EAE progression and neuroinflammation [77]
**miR-155**	Microglia Astrocytes Neurons	- Promotes pro-inflammatory activation of microglia and impairs myelin phagocytosis [78,79] - Expression in astrocytes releases microglia of inhibitory control of phagocytosis [80] - May be neuroprotective via the Nogo pathway [81,82]
**miR-30a**	Microglia	- Overexpression promotes release of factors that induce OPC apoptosis [83]
**miR-145**	Microglia Astrocytes	- Strongly associated with anti-inflammatory microglia activation [84] - Inhibits astrogliosis [32]
**miR-771**	Microglia	- Strongly associated with anti-inflammatory microglia activation [84]
**miR-99a**	Astrocytes	- Decrease increases astrocyte proliferation [32]
**miR-143**	Astrocytes	- Decrease increases astrocyte proliferation [32]
**miR-449**	Astrocytes	- Attenuates glial scar formation [32]
**miR-34a**	Astrocytes	- Expression in astrocytes releases microglia of inhibitory control of phagocytosis [80]
**miR-326**	Astrocytes	- Expression in astrocytes releases microglia of inhibitory control of phagocytosis [80]
**miR-20a**	Neurons	- May be neuroprotective via the Nogo pathway [81]
**miR-181a**	T cells Macrophages	- Promotes Treg differentiation [85] - Inhibits Th1 differentiation and pro-inflammatory macrophage polarisation [85]
**miR-27**	T cells	- Impairs Treg differentiation and Treg immunosuppressive activity [86]
**miR-132/212 cluster**	T cells	- Suppression inhibits Th1/Th17 differentiation without interfering with Treg differentiation [87]
**miR-106b/25 cluster**	T cells	- Possibly impairs Treg suppressor function by interfering with the TGF-β pathway [88]
**miR-665**	Oligodendrocytes	- Impairs OPC differentiation when induced by CD49d^+^CD154^+^ lymphocytes [89]
